# Overcoming taxonomic challenges in DNA barcoding for improvement of identification and preservation of clariid catfish species

**DOI:** 10.5808/gi.23038

**Published:** 2023-09-27

**Authors:** Piangjai Chalermwong, Thitipong Panthum, Pish Wattanadilokcahtkun, Nattakan Ariyaraphong, Thanyapat Thong, Phanitada Srikampa, Worapong Singchat, Syed Farhan Ahmad, Kantika Noito, Ryan Rasoarahona, Artem Lisachov, Hina Ali, Ekaphan Kraichak, Narongrit Muangmai, Satid Chatchaiphan, Kednapat Sriphairoj, Sittichai Hatachote, Aingorn Chaiyes, Chatchawan Jantasuriyarat, Visarut Chailertlit, Warong Suksavate, Jumaporn Sonongbua, Witsanu Srimai, Sunchai Payungporn, Kyudong Han, Agostinho Antunes, Prapansak Srisapoome, Akihiko Koga, Prateep Duengkae, Yoichi Matsuda, Uthairat Na-Nakorn, Kornsorn Srikulnath

**Affiliations:** 1 Animal Genomics and Bioresource Research Unit (AGB Research Unit), Faculty of Science, Kasetsart University, 50 Ngamwongwan, Chatuchak, Bangkok 10900, Thailand; 2 Sciences for Industry, Faculty of Science, Kasetsart University, 50 Ngamwongwan, Chatuchak, Bangkok 10900, Thailand; 3 Special Research Unit for Wildlife Genomics (SRUWG), Department of Forest Biology, Faculty of Forestry, Kasetsart University, 50 Ngamwongwan, Chatuchak, Bangkok 10900, Thailand; 4 Department of Botany, Faculty of Science, Kasetsart University, Bangkok 10900, Thailand; 5 Department of Fishery Biology, Faculty of Fisheries, Kasetsart University, Bangkok 10900, Thailand; 6 Department of Aquaculture, Faculty of Fisheries, Kasetsart University, Bangkok 10900, Thailand; 7 Faculty of Natural Resources and Agro-Industry, Kasetsart University Chalermphrakiat Sakon Nakhon Province Campus, Sakon Nakhon 47000, Thailand; 8 School of Agriculture and Cooperatives, Sukhothai Thammathirat Open University, Nonthaburi 11120, Thailand; 9 Department of Genetics, Faculty of Science, Kasetsart University, Chatuchak, Bangkok, 10900, Thailand; 10 Pathum Thani Aquatic Animal Genetics Research and Development Center, Aquatic Animal Genetics Research and Development Division, Department of Fisheries, Pathum Thani 12120, Thailand; 11 Faculty of Interdisciplinary Studies, Khon Kaen University, Nong Kom Ko, Mueang Nong Khai District, Nong Khai 43000, Thailand; 12 Kalasin Fish Hatchery Farm (Betagro), Buaban, Yangtalad district, Kalasin 46120, Thailand; 13 Research Unit of Systems Microbiology, Department of Biochemistry, Faculty of Medicine, Chulalongkorn University, Bangkok 10330, Thailand; 14 Department of Microbiology, Dankook University, Cheonan 31116, Korea; 15 Bio-Medical Engineering Core Facility Research Center, Dankook University, Cheonan 31116, Korea; 16 CIIMAR/CIMAR, Interdisciplinary Centre of Marine and Environmental Research, University of Porto, Terminal de Cruzeiros do Porto de Leixões, Av. General Norton de Matos, s/n, 4450-208 Porto, Portugal; 17 Department of Biology, Faculty of Sciences, University of Porto, Rua do Campo Alegre, s/n, 4169-007 Porto, Portugal; 18 Center for Agricultural Biotechnology, No. 1, Moo 6, Kamphaeng Saen, Kamphaeng Saen, Nakhon Pathom 73140, Thailand

**Keywords:** barcoding gap, catfish, public repository, sequence divergence, species delimitation

## Abstract

DNA barcoding without assessing reliability and validity causes taxonomic errors of species identification, which is responsible for disruptions of their conservation and aquaculture industry. Although DNA barcoding facilitates molecular identification and phylogenetic analysis of species, its availability in clariid catfish lineage remains uncertain. In this study, DNA barcoding was developed and validated for clariid catfish. 2,970 barcode sequences from mitochondrial cytochrome c oxidase I (*COI*) and cytochrome b (*Cytb*) genes and D-loop sequences were analyzed for 37 clariid catfish species. The highest intraspecific nearest neighbor distances were 85.47%, 98.03%, and 89.10% for *COI*, *Cytb*, and D-loop sequences, respectively. This suggests that the *Cytb* gene is the most appropriate for identifying clariid catfish and can serve as a standard region for DNA barcoding. A positive barcoding gap between interspecific and intraspecific sequence divergence was observed in the *Cytb* dataset but not in the *COI* and D-loop datasets. Intraspecific variation was typically less than 4.4%, whereas interspecific variation was generally more than 66.9%. However, a species complex was detected in walking catfish and significant intraspecific sequence divergence was observed in North African catfish. These findings suggest the need to focus on developing a DNA barcoding system for classifying clariid catfish properly and to validate its efficacy for a wider range of clariid catfish. With an enriched database of multiple sequences from a target species and its genus, species identification can be more accurate and biodiversity assessment of the species can be facilitated.

## Introduction

Over 3,000 species, 478 genera, and 36 families of catfish have been identified; these fish represent an important global protein source. In particular, the yield of clariid catfish (*Clarias*) reached 94,277 tons in 2022 in Thailand [1]. Clariid species, such as walking catfish (*Clarias batrachus*), bighead catfish (*C. macrocephalus*), and whitespotted clarias (*C. fuscus*), are common in the global aquaculture market [2-6]. However, their habitat loss, overfishing, and introduction of North African catfish (*C. gariepinus*) through various trade channels have significantly been decreasing the populations of walking and bighead catfish. Introduction of North African catfish for improving production, such as hybridization by producing hybrids with other species, negatively affects native fish fauna via predation and hybridization [4,6,7]. This exotic species also poses a risk of genetic contamination and displacement of indigenous species if it escapes into the wild. Countries such as India and United States have banned the culture or import of North African catfish because of the potential threat posed by this fish on aquatic biodiversity; however, some fish farmers continue to raise it because of the ease of rearing [7,8].

Diverse sources of clariid catfish and misidentification of certain species, such as whitespotted clarias, make it difficult to classify individual species precisely. Morphological character-based classification methods are insufficient and remain controversial due to morphologies's similarity among clariid catfish [2,9,10]. Identification based on fish morphology information obtained from the FishBase (http://www.fishbase.org) is limited by the impact of living conditions or processed products, rendering classification difficult or even impossible. New fish species have been described using molecular approaches that overcome the challenges associated with morphology-based identification [11,12]. DNA barcoding goes through a set of process of sequencing a short segment of a DNA barcode of unknown species, comparing it with the data in a barcode database of known species, and thereby providing an alternative to morphology-based identification [4,13,14]. DNA barcoding has revealed hidden species diversity in many organisms. However, its ability to delineate species in the clariid catfish lineage remains uncertain [2,15]. The accurate clustering of species is crucial because genetic distance can differ across lineages. Public repositories currently contain numerous nucleotide sequences of clariid catfish from many studies. The status of clariid species in different regions/localities has been disputed, and their actual species richness may have been overestimated. Walking catfish specimens in Southeast Asia have a genetic distance of 0.78% from those in India, whereas those within Southeast Asia differ by 6.98% [4]. More specimens and localities are required to improve the quality of the nucleotide public repository (NIH genetic sequence database [GenBank]/DNA Data Bank of Japan [DDBJ]/European Nucleotide Archive database [ENA]), prevent errors of species identification, and address the issue of erroneous sequences resulting from misidentification, and contamination. Misidentification can cause large intraspecific sequence divergence, and the mislabeling of DNA sequences in clariid catfish repositories, such as for North African catfish, has been reported [2,4,14]. Barcodes can be reexamined by providing more information to resolve potentially conflicting claims. To ensure the accuracy and reliability of barcode system, the ambiguity of nucleotide barcode sequences in reference libraries should be investigated.

A quality check of available nucleotide sequences of clariid catfish in public repositories is necessary to establish an international standard for database management [13,16]. Here, it was hypothesized that sequence errors in public repositories lead to misidentification of species owing to discrepancies between the nomenclature and DNA barcodes. This study aimed to (1) differentiate between clariid catfish species using DNA barcoding and (2) confirm previous DNA barcoding findings [2,3,15]. To improve the accuracy of species identification, available clariid catfish sequences were analyzed for potential errors using sequence divergence analyses of mitochondrial cytochrome c oxidase I (*COI*) and cytochrome b (*Cytb*) genes and D-loop sequences, for which large sequence accessions have been deposited in the public repository of clariid catfish. Species identities were validated using nucleotide barcodes and species delimitation analyses, which revealed high accuracy.

## Methods

### Acquisition of partial mitochondrial clariid catfish nucleotide sequences

Partial mitochondrial nucleotide sequences of clariid catfish were acquired from public repositories, with focus on the *COI*, *Cytb*, and D-loop sequences, which are frequently used for identification of clariid catfish species and their phylogenetic analysis [3,13,15,17-20]. Each dataset was subjected to multiple sequence alignments using Geneious Prime (version 2020.0.3). To avoid the risk of nuclear mitochondrial DNA contamination, sequences with deletions or gaps were also included in the analysis. Although the sequence lengths varied, the most frequently occurring sequence lengths were chosen for further examination [21]. The selected *COI* and *Cytb* coding sequences were translated into the amino acid sequences using Molecular Evolutionary Genetics Analysis version X (MEGA-X) [22] and aligned to ensure the presence of an open reading frame without a stop codon [23].

### Data analysis

The alignment length, variable positions, and GC content were compared among the *COI*, *Cytb*, and D-loop sequence datasets. Substitution saturation was evaluated by plotting the number of transitions (s) and transversions (v) against Kimura 2-parameter (K2P) sequence divergences and comparing the information entropy-based index (I_ss_) with critical values (I_ss.c_) [24-26], as implemented in DAMBE [27]. If I_ss_ is significantly lower than I_ss.c_, then substitution saturation does not occur in the sequences [27]. Nucleotide substitutions at the third codon position and those at the first and second codon positions were also tested separately for each dataset. Sequence divergence was used to evaluate the discriminatory power of distance- and tree-based approaches. The nearest neighbor test [28] was applied to determine whether the nearest neighbor was intraspecific and to assess whether a sufficient gap occurred between the intraspecific and interspecific sequence divergences. Sequence divergence among individual sequences was calculated using the K2P model, which is a standard model used in barcoding studies. The function dist.dna in the R package "ape" was used for examination in the distance-based approach [29-32]. To identify whether sequences with the shortest divergences were the same, the function "nearNeighbor" in the R package "spider" was used to perform the nearest neighbor test [31]. The percentage of correctly identified sequences was computed by dividing the number of sequences with an intraspecific nearest neighbor by the total number of sequences. The barcoding gap was determined by calculating the difference between the minimum and maximum intraspecific sequence divergences using the “non-ConDist” and “maxInDist” functions in the R package “spider” [31]. Barcoding gaps were evaluated by applying the Kruskal-Wallis test to determine whether there were significant differences between the *COI*, *Cytb*, and D-loop sequence datasets [33]. To assess significant differences, Dunn’s test with Bonferroni correction was used for multiple comparisons [34]. Markers with high discriminatory power were identified by selecting those with a high percentage of correct identifications from the nearest neighbor test and a positive value for the barcoding gap.

Phylogenetic analyses were carried out using Bayesian inference (BI) methods in MrBayes version 3.2.6 to examine the *COI*, *Cytb*, and D-loop sequence datasets using a tree-based approach [35]. Wide-head catfish (*Clarotes laticeps*) was used as the outgroup for the *COI* and *Cytb* datasets (GenBank accession number OM176590 for *COI* [36]; and GenBank accession number HG803407 for *Cytb* [37]), whereas *Pseudobagrus taeniatus* was used as the outgroup for the D-loop dataset (GenBank accession number AB097696 [38]). Four chains were run simultaneously for two million generations, with sampling every 1,000 generations, using the Markov chain Monte Carlo process. The burn-in period was discarded before convergence was reached, and the Bayesian posterior probability was obtained as a percentage of the sampled tree population. The BI tree was visualized using FigTree version 1.4.4. The number of monophyletic groups was calculated with the R package “spder” using the “monophyly” function [31]. The tree-based test for barcoding efficiency aimed to assess a marker's ability to recover monophyly among sequences of the same species without using relationships among the studied taxa as a criterion, although phylogenetic reconstruction with a small dataset may result in poorly resolved relationships among species and be typically avoided in systematic studies [16,29,31].

### Species delimitation

The delimitation of clariid catfish species was assessed using two approaches: the Bayesian Poisson tree process (bPTP) method [39] and the General Mixed Yule Coalescent (GMYC) method [40]. All three datasets were tested separately. For the bPTP method, a Bayesian implementation of the PTP model was used with the maximum likelihood tree as the input file via the PTP web server (http://species.h-its.org) using the default settings. An ultrametric tree was constructed using the "chronos" function in the R program and used as input for the GMYC analyses. The GMYC delimitation method with a single threshold model was performed using the "gmyc" function in the R package "splits" (R-Forge, http://r-forge.rproject.org/projects/splits/).

### Data availability statement

The full dataset and metadata from this study are available from the Dryad Digital Repository (data set: https://datadryad.org/stash/share/JTJgvoR1X35SeML9B7A2H0BFaHRKPTz0JorqYW8BJyw).

### Ethical approval

All animal care and experimental procedures were approved (approval no. ACKU65-SCI-003 and ACKU65-SCI-026) by the Animal Experiment Committee of Kasetsart University, and conducted in accordance with the Regulations on Animal Experiments at Kasetsart University.

## Results

### Available data for analysis

The nucleotide sequences of mitochondrial *COI*, *Cytb*, and D-loop region of clariid catfish were compiled to test whether DNA barcoding could identify erroneous sequences in public repositories. Their sequence lengths were varied, and a tradeoff was observed between maximum of alignment length and taxonomic coverage. To address intraspecific variability, most species were represented by multiple specimens, with an average of 75 specimens per species. However, the data of only one specimen were available for 14 species, including 6 in the *COI* dataset (walking catfish [*C*. aff. *Batrachus*], Alluaud's catfish [*C. alluaudi*], *C. buettikoferi*, *C. platycephalus*, *C. teijsmanni*, and Werner's catfish [*C. werneri*]), 9 in the *Cytb* dataset (walking catfish [*C*. aff. *batrachus*], *C. buettikoferi*, *C. camerunensis*, *C. ebriensis*, *C. jaensis*, *C. microstomus*, *C. planiceps*, *C. pseudoleiacanthus*, and snake catfish [*C. theodorae*]), and 2 in the D-loop sequence dataset (walking catfish [*C*. aff. *batrachus*] and blackskin catfish [*C. meladerma*]). The nucleotide sequence database contained 2,970 barcoding sequences from 3,026 accession number sequences of clariid catfish, including 670 *COI* sequences (710 bp), 782 *Cytb* sequences (510 bp), and 1,518 D-loop sequences (593 bp). No stop codons or frameshift mutations were found in any of the sequences, indicating that they were all partial *COI* and *Cytb* genes fragments. The dataset ultimately included with varying base pairs and 346, 138, and 420 bp of parsimonious informative sites, respectively.

### Sequence divergence and distance-based evaluation

**Fig. 1.**
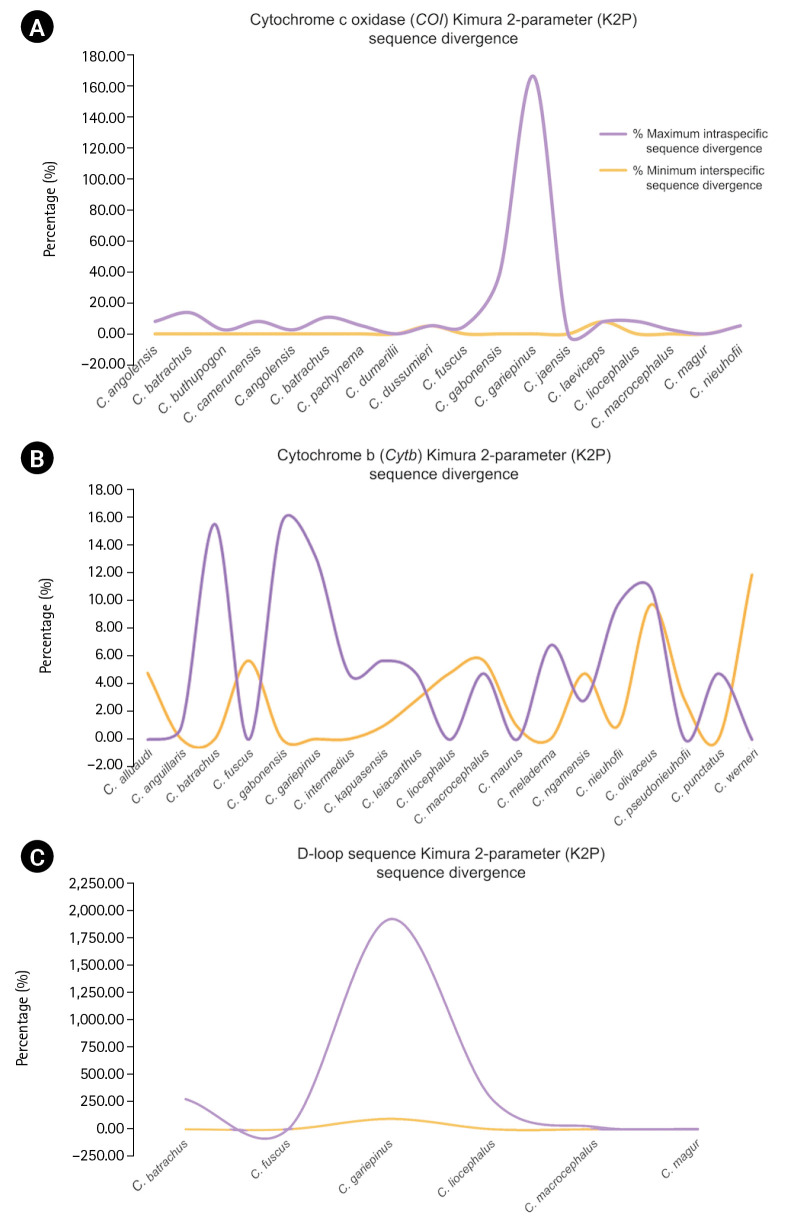
Distribution of maximum intraspecific (red triangle) and minimum interspecific (blue square box) uncorrected pairwise distances and K2P sequence divergence of 18 species based on *COI*, 19 species based on (*Cytb*), and 6 species based on mitochondrial D-loop sequence. Dot plot analysis of *COI* (A), *Cytb* (B), and D-loop (C) sequence divergence. The abbreviations of species name on the x-axis are listed in Table 2. The y-axis indicates the level of sequence divergence shown as K2P sequence divergence. Sequence divergences of the species are represented by more than one sample.

**Fig. 2.**
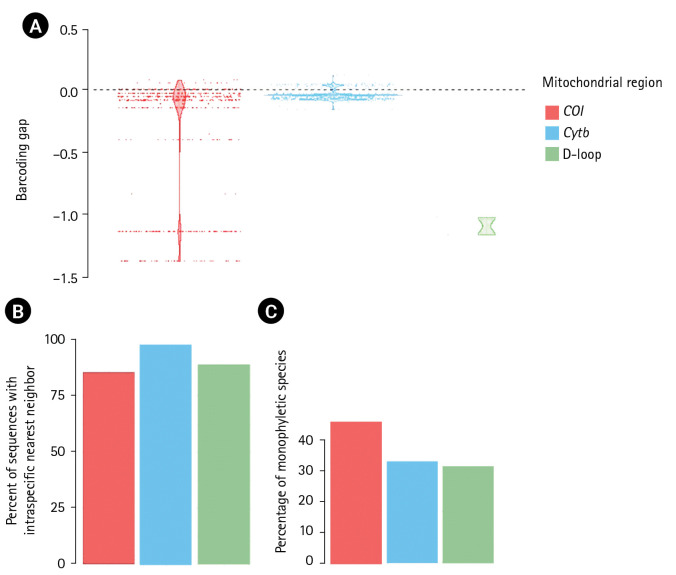
Distance-based comparison of efficiency among the cytochrome c oxidase I (*CO1*), cytochrome b (*Cytb*), and D-loop sequence barcodes of clariid catfish species from the database. (A) Distribution of barcoding gaps, which are defined by the difference between minimum interspecific distance and maximum intraspecific distance. (B) Percentage of correct identifications estimated by the nearest neighbor test. (C) A tree-based comparison of efficiency among the studied barcoding markers for clariid catfish species from the database using the percentage of monophyletic groups recovered between *CO1, Cytb*, and D-loop sequence datasets.

The alignment length, number of variable sites, and GC content and intraspecific sequence divergence are shown in Table 1. In the *COI* dataset, the minimum intraspecific K2P sequence divergence was 0% for *C. dumerilii*, Valenciennes clariid (*C. dussumieri*), *C. jaensis*, mudfish (*C. laeviceps*), *C. magur*, and slender walking catfish (*C. nieuhofii*). The maximum intraspecific K2P sequence divergence was 133.09% ± 23.40% in North African catfish (*C. gariepinus*). The minimum interspecific K2P sequence divergence was 0% in Angolian walking catfish (*C. angolensis*), *C. dumerilii*, whitespotted clarias (*C. fuscus*), *C. jaensis*, smoothhead catfish (*C. liocephalus*), and *C. magur*. The maximum interspecific K2P sequence divergence was 7.90% ± 0.00% in mudfish (*C. laeviceps*). For the *Cytb* dataset, the minimum intraspecific K2P sequence divergence was 0% in Alluaud catfish, whitespotted clarias (*C. fuscus*), smoothhead catfish (*C. liocephalus*), *C. maurus*, *C. pseudonieuhofii*, and Werner's catfish (*C. werneri*). The maximum intraspecific K2P sequence divergence was 14.97% ± 0.00% in *C. gabonensis*. The minimum interspecific K2P sequence divergence was 0.26% ± 0.60% in *C. punctatus*. The maximum interspecific K2P sequence divergence was 11.31% ± 0.00% in Werner’s catfish (*C. werneri*) (Table 2). For the D-loop sequence dataset, the minimum intraspecific K2P sequence divergence was 1.19% ± 0.00% in whitespotted clarias (*C. fuscus*). The maximum intraspecific K2P sequence divergence was 469.46% ± 578.35% in North African catfish (*C. gariepinus*). The minimum interspecific K2P sequence divergence between North African catfish and other clariid catfish was 110.63% ± 19.58%. The maximum interspecific K2P sequence divergence was 124.28% ± 19.58% for North African catfish. The maximum and minimum interspecific sequence divergences were used to establish a barcoding gap for species identification (Fig. 1). The interspecific sequence divergence in the *Cytb* dataset tended to be greater than the intraspecific one, thus leading to a positive barcoding gap. Most inter- and intraspecific sequence divergences were likely nonzero, which was evident from the distribution of the barcoding gaps. By contrast, in the *COI* and D-loop sequence datasets, a negative barcoding gap was observed, and the intraspecific sequence divergences were mainly greater than the interspecific sequence divergences (Fig. 2A). The three datasets had significantly different barcoding gaps (Kruskal-Wallis’s test, *χ*^2^ = 183.01, p < 0.01). According to the pairwise comparison, the *Cytb* barcoding gap was significantly different from those of *COI* (Z = –13.38, p < 0.01) and D-loop gaps (Z = 2.44, p < 0.05). By contrast, no significant differences were observed between the *COI* and D-loop sequence datasets (Z = 1.43, p = 0.46). The *COI*, *Cytb*, and D-loop sequences had 85.47%, 98.03%, and 89.10% of intraspecific nearest neighbor distances, respectively (Fig. 2B).

### Phylogenetic analyses and tree-based evaluation

Reconstruction of phylogenetic relationships of clariid catfish using the *COI*, *Cytb*, and D-loop sequence datasets strongly supported that 296 specimens belonged to a single clariid catfish species with a high posterior probability. The highest percentage of monophyletic groups (42%) was observed in the phylogenetic tree of the *COI* sequence dataset, whereas lower percentages of monophyletic groups (30% and 28%) were observed in the phylogenetic trees of the *Cytb* and D-loop sequence datasets, respectively (Fig. 2C).

### Species delimitation with the *COI*, *Cytb*, and D-loop sequence datasets

The bPTP and GMYC methods were used to delimit the species using the *COI*, *Cytb*, and D-loop sequence datasets. The bPTP method supported 86, 141, and 1,289 species for the *COI*, *Cytb*, and D-loop sequence datasets, respectively. The GMYC method supported 68 and 21 species for the *COI* and *Cytb* sequence datasets, respectively; however, the D-loop sequence dataset was not included because data were not available to delimit the species. (Supplementary Figs. 1–3).

### Substitution saturation

**Fig. 3.**
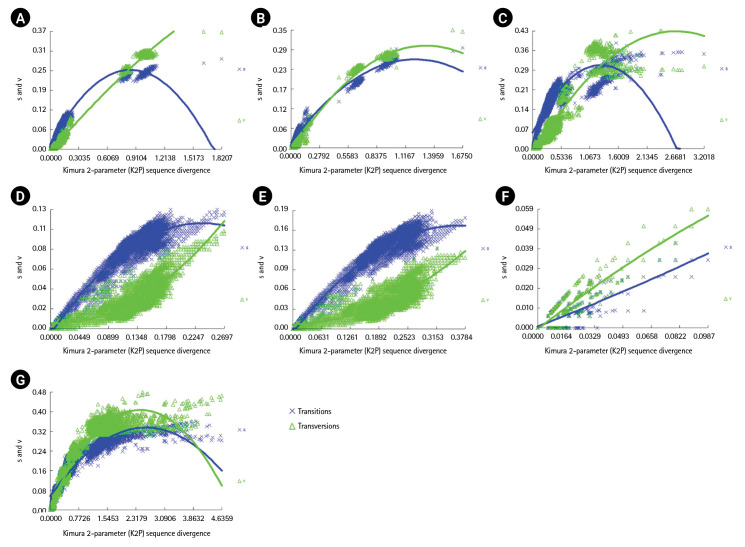
DAMBE substitution saturation plots for the sequences of whole cytochrome c oxidase I (*COI*) region (A), *COI* codon positions 1 and 2 (B), *COI* codon position 3 (C), whole cytochrome b (*Cytb*) region (D), *Cytb* codon positions 1 and 2 (E), and *Cytb* codon position 3 (F); and whole D-loop region (G). The number of transitions (s) and transversions (t) is plotted against the K2P distance. Mean values thick lines) and standard deviations (fine lines) of the transitions (s) and transversions (v) are shown.

The *COI*, *Cytb*, and D-loop sequence datasets were assessed for used for assessment of substitution saturation. The number of mutations, including transitions and transversions, was higher in the *Cytb* sequence dataset than the *COI* and D-loop sequence datasets. In the *Cytb* sequence dataset, a linear correlation was observed between the number of transitions and transversions when plotted against the sequences (Fig. 3). The I_ss_ values were lower than the I_ss.c_ values in the *COI* and *Cytb* sequence dataset, whereas the I_ss_ values in the D-loop sequence dataset were higher than the I_ss.c_ values (Table 3).

## Discussion

Taxonomic identification of species has been a big challenge in clariid catfish because of inadequate descriptions of their morphologies and extensive plasticity [2,41-43]. DNA barcoding has helped to resolve some identification issues and determine the actual species composition. Although DNA barcoding provides additional important data for precise identification and classification of species, its availability is restricted on a limited number of reference libraries for sequence matching. Data interpretation can be complicated by variations at both the individual and population levels in diverse sampling areas. Expanding DNA barcode reference libraries is thus crucial for identifying questionable specimens.

As of April 2023, public repositories have amassed 59,304 nucleotide sequences from clariid catfish, and they include 3,748 mitochondrial and 55,556 nuclear sequences. Most of the nuclear sequences were obtained from functional gene analyses [44-47]. Over 5.11% of these nucleotide sequence resources contain *COI*, *Cytb*, and D-loop sequences, which are popular markers for molecular taxonomy and species identification of clariid catfish [3,13,15,17-20]. Here, “group 1,” which consists of 525 *COI* and 713 *Cytb* accessions, was correctly identified as the same species using distance-based evaluation. This result is consistent with the intraspecific nearest-neighbor percentages for *COI*, *Cytb*, and D-loop datasets. However, some *COI* and *Cytb* specimens (128 and 59, respectively) could not be used to differentiate between highly similar species because they showed conflicting results in the database, and they were classified as “group 2,” which exhibited higher-level similarity to multiple species. Moreover, intraspecific sequences divergences displayed interspecific sequence divergence, including *Clarias* sp., *C. batrachus*, *C. camerunensis*, *C. angolensis*, *C. fuscus*, and *C. liocephalus*, thus indicating a discrepancy between the nomenclature and DNA barcodes. This was evident in both public data repositories GenBank (https://www.ncbi.nlm.nih.gov/) and BOLD (https://www.boldsystems.org/). “Group 3” consisted of 11 unique *COI* sequences and 1 unique *Cytb* sequence that showed no similarity with most sequences under the same species name in public data repositories, such as *Clarias* sp., *C. batrachus*, *C. fuscus*, *C. camerunensis*, *C. angolensis*, and *C. liocephalus*. This suggests that the barcode reference data available for these species, which are obtained from public repositories, are insufficient. For validation of species identifications based on public repositories and BOLD, a reference database with at least three barcoded specimens for each species and a conspecific distance match of less than 1% are required, which is not applicable for the current clariid catfish barcodes in the *COI*, *Cytb*, and D-loop sequence dataset [48]. Hence, accurate species labeling, morphological taxonomy, and voucher documentation must be prioritized to re-evaluate spurious data. Alternatively, tree-based evaluation identified three classes, with “class 1” including sequences of the same species that exhibited both intraspecific clustering and distinct interspecific clustering with high probability (0.90–1.00), “class 2” including sequences of the same species with no intraspecific clustering, and “class 3” including sequences of different species, exhibiting cohesive clustering. Over 500 sequences in classes 2 and 3 were labeled as *Clarias* sp., Angolian walking catfish (*C. angolensis*), bighead catfish (*C. macrocephalus*), North African catfish (*C. gariepinus*), smoothhead catfish (*C. liocephalus*), Valenciennes clariid (*C. dussumieri*), walking catfish (*C. batrachus*), whitespotted clarias (*C. fuscus*), *C. buthupogon*, *C. camerunensis*, C. pachynema, *C. gabonensis*, and *C. magur* for *COI* and *Clarias* sp., bighead catfish (*C. macrocephalus*), blackskin catfish (*C. meladerma*), blunttooth catfish (*C. ngamensis*), North African catfish (*C. gariepinus*), slender walking catfish (*C. nieuhofii*), walking catfish (*C. batrachus*), whitespotted clarias (*C. fuscus*), *C. anguillaris*, *C. gabonensis*, *C. kapuasensis*, forest walking catfish (*C. leiacanthus*), *C. maurus*, *C. pseudonieuhofii*, and *C. punctatus* for *Cytb*, suggesting that species identifications in clariid catfish using these markers remain uncertain. However, most of species whose D-loop sequences were examined were categorized into classes 2 and 3, indicating that D-loop sequences are not applicable for identification of clariid catfish species, which is consistent with the findings for D-loop sequences in other vertebrates [49]. The success rate of DNA barcode identification in clariid catfish is relatively low (91%) compared to that in teleosts, due to the difficulty in detecting errors and confirming taxonomic accuracy or contamination [3,50]. In Falade et al. (2016) [14], 98%–100% of North African catfish were correctly identified. Misidentification can be effectively eliminated by combining morphological and distance-based algorithms, such as setting a threshold level for species identification and using clustering methods (Supplementary Figs. 4 and 5).

### Exploration of the barcoding gap in clariid catfish using distance-based evaluation

The analysis of nucleotide sequence divergence of the *COI* gene for more than 1,000 teleost species indicated that the probability of intraspecific sequence divergence was 3% or less [51]. However, some teleosts, such as mountain barbel (*Amphilius platychir*) and *Amphilius rheophilus*, have shown a cutoff score at the species level that may be influenced by the rate of mutation or the existence of cryptic species [52]. Low sequence divergence within species complex or even cryptic species has been predicted in clariid catfishes, such as walking catfish [19]. In the present study, the *Cytb* dataset showed no saturation in the saturation analyses, which is consistent with the positive barcoding gap, whereas the *COI* and D-loop sequence datasets showed saturation. The transition to transversion ratios of mitochondrial DNA in clariid catfish are similar to those in many teleosts with a larger excess of transitions compared to transversions [14]. The *Cytb* gene may be more informative in the clariid catfish lineage at the species level than the D-loop sequences that are appropriate for population studies, whereas the *COI* gene may not be fit for systematic studies of specific lineages [18]. Intraspecific sequence divergence is generally lower than interspecific sequence divergence. A threshold value can distinguish biological species based on nearest-neighbor sequence divergence. However, the evolutionary rate of mitochondrial DNA varies within and between species and also differs in its genomic regions, resulting in a broad overlap of intra- and interspecific distances [53]. To improve the efficiency of precise species identification, the approach for examining the maximum intraspecific sequence divergence versus minimum interspecific sequence divergence resulted in making a barcoding gap four times as small compared with that reported in previous fish DNA barcoding studies [2]. In the present study, simplifying the data by ignoring complex groups, such as walking catfish and North African catfish, led to a barcoding gap for the *Cytb* dataset that was –0.15 to 0.11 for the species identification of clariid catfish, while the gaps of the *COI* and D-loop sequence datasets were –1.64 to 0.08 and –1.12 to –0.98, respectively. These values are consistent with the average barcoding gap of 83.6% reported in studies in other fish [13,20,54]. This suggests that the clariid catfish exhibited high levels of intraspecific sequence divergence. However, the different results were obtained for walking catfish from the Philippines and India, in which large barcoding gaps were detected for the *COI* gene [13,15]. Mislabeling of the species may have occurred because of the large genetic distance between populations of walking catfish in each the Philippines and India. Additionally, the Indian species, *C. magur*, may have been mistaken for walking catfish because of differences in head shape and pectoral spine serration from those found in Southeast Asia [55]. Karyotypic differences have also been observed between walking catfish populations in India and Thailand, indicating their genetic dissimilarity [55]. The presence of geographically divergent populations of walking catfish could explain the low or unmatched results for walking catfish specimens. This suggests that the low or unmatched results for walking catfish specimens may be due to the presence of geographically divergent populations. Moreover, artificial hybridization of bighead catfish and walking catfish for aquaculture purposes is becoming more popular; however, reports have indicated that distinguishing between female walking catfish and bighead catfish remains challenging [15].

Large intraspecific sequence divergences were observed in North African catfish, which were more than expected in the present study. This may be caused by the genetic difference between domesticated strains of the same species. Nonetheless, all specimens were classified as belonging to the same species, using the bPTP and GMYC methods. The ZZ/ZW sex determination system is believed to be the same as the ancestral system for North African catfish, which is supported by most of the reported sex chromosome systems from Africa and Israel. However, some studies of different populations from Israel, Hungary, and China suggest an XX/XY system or the possible coexistence of both sex chromosome systems or polygenic sex determination in North African catfish [56-62]. Unlikely hypotheses of incomplete lineage sorting in North African catfish can be ruled out if (1) populations with highly divergent mitochondrial DNA haplotypes are present in the same species or (2) the same haplotype are accumulated geographically near the boundaries of allopatric species or in their hybrid zones. It should be noted that haplotype retained from a common ancestor should be randomly distributed in the populations of descendant species, not accumulated near the boundaries of allopatric species or in their hybrid zones. This suggests that the problems in taxonomic and systematic analyses have complex issues that need to be resolved based on the data obtained from populations from different geographic origins and by large-scale genomic analyses [63,64].

The *COI* and D-loop sequence datasets had lower power than the *Cytb* dataset because they had fewer informative sites. The overlap in the distribution of intra- and interspecific sequence divergence resulted in unclear cutoff values for species identification. The paradox of deep intraspecific and shallow interspecific sequence divergence indicates the need for further verification of specimen collection accuracy. However, species identification based on the *Cytb* dataset was unambiguous for Alluaud's catfish (*C. alluaudi*), whitespotted clarias (*C. fuscus*), North African catfish (*C. gariepinus*), *C. intermedius*, smoothhead catfish (*C. liocephalus*), bighead catfish (*C. macrocephalus*), blunttooth catfish (*C. ngamensis*), slender walking catfish (*C. nieuhofii*), *C. olivaceus*, *C. pseudonieuhofii*, and Werner's catfish (*C. werneri*). The optimal quality and traceability of the data associated with reference barcodes must be ensured by developing and adhering to best practices. To build a robust reference sequence library in public repositories, specimens from various geographical locations are required improvement of the *Cytb* barcoding gap value will provide more reliable representative data. However, cryptic species complexes in clariid catfish lineages may cause an overlap intraspecific and interspecific sequence divergences.

### Detection of cryptic clariid catfish: one aspect of the issue

DNA barcoding and species delimitation methods can reveal the existence of cryptic species among known species, thus providing an objective means of testing evolutionary independence. The integration of these methods is crucial for accurately determining regional biodiversity, and the consistent results obtained through all means have confirmed the boundary of species. Compared the results of sequence divergence with species delimitation in North African catfish, one individual was clustered away from walking catfish and whitespotted clarias individuals, with divergence in the interspecific range (group 3 and class 3) and clear cutoff intervals, using the two specific delimitation methods (the bPTP and GMYC methods). Whether this sequence belongs to a different cryptic species or represents a distant gene pool within the same species remains unclear. This individual may indicate a different ecotype with a diverse geographical location or may have resulted from a species identification error by observation. Such high intraspecific sequence divergence has also been reported previously in haiwels (*Pangasius macronema*) and striped catfish (*Pangasianodon hypophthalmus*), resulting from geographical isolation and substantial habitat reorganization [50]. Intraspecific sequence divergence between these two species was likely overlapped with the interspecific sequence divergence, and significantly different genetic or population structures cannot be ruled out, particularly in small-sized samples [65]. Our results showed that mismatches between nomenclature and barcode by sequence errors of clariid catfish in repositories is most probable cause of the existence of cryptic species complexes due to misidentification of species. The probability of discovering a new species has already been reported in a molecular study of clariid catfish [11], which identified one unknown lineage which were from Lake Tanganyika, and they were included among the previously identified six species identified previously. This also indicates that the diversity within *Pseudotanganikallabes prognatha* (new genus and species of clariid catfish) from *Clarias* might be larger than that detected by morphology. This raises the question of whether *Pseudotanganikallabes* and *Clarias* belong to the same genus with high intraspecific genetic variability or multiple species. Such ambiguously identified species often form species complexes when close examination is carried out for specimen collected from broad geographicareas [66]. A thorough examination of the genetically diverged groups of *C. gabonensis* in our dataset is required to determine the presence of new species.

### Problems of identifying species using certain genera and database

The BOLD identification system and a public data repository for BLASTN comparison of nucleotide sequences were created for different purposes and operated differently [48,67]. BOLD was designed for taxonomic identification using a limited number of loci and similarity thresholds to match query sequences with high confidence and to identify sequences in the database. By contrast, BLASTN was developed to assess sequence similarity and the most similar sequences in public data repositories [68]. However, misidentified sequences are present in both BOLD and public data repositories, thereby complicating DNA-based identification. The accuracy of the sequence data cannot be confirmed because sequence trace files or voucher samples are not available through public data repositories. Similarly, the BOLD database faces challenges in verifying suspected records despite efforts made to improve quality control. Erroneous identification of species by sample sequences can occur when applying BOLD to species with wrong information registered in public data repositories. Additionally, a significant proportion of barcodes available for BOLD are sourced from public repositories, which may contain a large number of questionable, erroneous, or low-quality sequence data owing to a dramatic increase of registered sequence information along with the development of high throughput sequencing technologies [69]. The walking catfish complex is an example of a case in which mislabeling of accession sequences from bighead catfish, or accidental utilization of interspecific hybridization in cataloging the barcode database, may have occurred. To ensure the accuracy of species identification by DNA barcoding users should prioritize correct species labeling reliable morphological taxonomy, and voucher documentation.

The lack of accurate taxonomic and genetic information has caused misidentification of clariid catfish, resulting in hindering the monitoring of habitat changes caused by habitat loss, overfishing, and non-native species introductions, which is prerequisite for planning their conservation and aquaculture. The limitations of BOLD and present public repositories for clariid catfish, where sequences from potentially misidentified specimens were contained, were revealed in this study. Thus, taxonomic uncertainties in challenging morphological groups and similar situations can be resolved. Therefore, the validated DNA barcode sequence library of clariid catfish obtained in this study can serve as a reference for examining species boundaries among closely related taxa, which is required for planning conservation strategies and increasing aquaculture productivity. Mitochondrial *Cytb* gene barcoding is highly effective for identifying clariid catfish precisely because it is reliable and informative with a large dataset of nucleotide sequences. Thus, guidelines for international standards and digital infrastructure to manage genetic resources of clariid catfish have been introduced, which brings out the full potential of biological resource stored in public nucleotide repositories. Future studies should prioritize barcoding for specific clariid catfish groups, such as walking catfish or North African catfish, which have high utility value for aquaculture production, and validate the efficacy of the marker as a barcoding region for a broader selection of species. A comprehensive DNA barcode library can also aid in identifying new species and improving our understanding of endemic clariid catfish resources.

## Figures and Tables

**Table 1.** List of markers used for studies in clariid catfish

Marker	Alignment length (bp)	Variable site	GC content (%)
*COI*	710	588	44.12
*Cytb*	510	285	43.94
D-loop	593	552	32.22

*COI*, cytochrome c oxidase I; *Cytb*, cytochrome b.

**Table 2.** Comparison of Kimura’s-two-parameter sequence divergence between individual sequences from the public repositories

Species	Common name	*COI*	*Cytb*	D-loop
*Clarias alluaudi*	Alluaud's catfish	–	0.000	–
*Clarias angolensis*	Angolian walking catfish	0.053–0.081	–	–
*Clarias anguillaris*	–	–	0.009–0.009	–
*Clarias batrachus*	Walking catfish	0.080–0.138	0.094–0.148	0.130–2.783
*Clarias buettikoferi*	–	–	–	–
*Clarias buthupogon*	–	0.026–0.026	–	–
*Clarias camerunensis*	–	0.081–0.081	–	–
*Clarias dumerilii*	–	0.000	–	–
*Clarias dussumieri*	Valenciennes clariid	0.000	–	–
*Clarias ebriensis*	–	–	–	–
*Clarias fuscus*	Whitespotted clarias	0.052–0.052	0.000	0.012–0.012
*Clarias gabonensis*	–	0.343–0.384	0.150–0.150	–
*Clarias gariepinus*	North African catfish	1.122–1.671	0.064–0.126	1.978–19.339
*Clarias intermedius*	–	–	0.027–0.045	–
*Clarias jaensis*	–	0.000	–	–
*Clarias kapuasensis*	–	–	0.045–0.054	–
*Clarias laeviceps*	Mudfish	0.000	–	–
*Clarias leiacanthus*	Forest walking catfish	–	0.027–0.045	–
*Clarias liocephalus*	Smoothhead catfish	0.081–0.081	0.000	0.128–2.691
*Clarias macrocephalus*	Bighead catfish	0.026–0.026	0.027–0.045	0.158–0.225
*Clarias magur*	–	0.000	–	0.012–0.024
*Clarias maurus*	–	–	0.000	–
*Clarias meladerma*	Blackskin catfish	–	0.036–0.065	–
*Clarias microstomus*	–	–	–	–
*Clarias ngamensis*	Blunttooth catfish	–	0.018–0.027	–
*Clarias nieuhofii*	Slender walking catfish	0.000	0.064–0.093	–
*Clarias olivaceus*	–	–	0.083–0.104	–
*Clarias planiceps*	–	–	–	–
*Clarias platycephalus*	–	–	–	–
*Clarias pseudoleiacanthus*	–	–	–	–
*Clarias pseudonieuhofii*	–	–	0.000	–
*Clarias punctatus*	–	–	0.036–0.045	–
*Clarias* sp.	–	–	–	–
*Clarias teijsmanni*	–	–	–	–
*Clarias theodorae*	Snake catfish	–	–	–
*Clarias werneri*	Werner's catfish	–	0.000	–

*COI*, cytochrome c oxidase I; *Cytb*, cytochrome b; –, no data available in the database.

**Table 3.** Substitution saturation analyses of *COI*, *Cytb*, and D-loop sequence datasets are based on the index of substitution saturation, as implemented in DAMBE [27]

Region	No. of OTUs^a^	I_ss_^b^	I_ss.cSym_^c^	df^d^	p-value^e^	I_ss.cAsym_^f^	df	p-value
*COI*	4	0.323	0.808	705	0	0.777	705	0.000
	8	0.355	0.769	705	0	0.660	705	0.000
	16	0.383	0.749	705	0	0.541	705	0.000
	32	0.439	0.723	705	0	0.399	705	0.436
*Cytb*	4	0.215	0.796	509	0	0.762	509	0.000
	8	0.207	0.752	509	0	0.641	509	0.000
	16	0.214	0.722	509	0	0.513	509	0.000
	32	0.252	0.703	509	0	0.378	509	0.000
D-loop	4	0.988	0.800	563	0	0.767	563	0.000
	8	1.112	0.758	563	0	0.647	563	0.000
	16	1.288	0.732	563	0	0.521	563	0.000
	32	1.490	0.709	563	0	0.381	563	0.000

*COI*, cytochrome c oxidase I; *Cytb*, cytochrome b.

a Number of sequences used for random resampling; OTU, operational taxonomic unit.

b Index of substitution saturation.

c Critical value for a symmetrical tree topology.

d Degrees of freedom.

e Probability that I_ss_ is signifcantly diferent from the critical value (I_ss.cSym_/I_ss.cAsym_).

f Critical value for an asymmetrical tree topology.

## Supplementary Materials

Supplementary Fig. 1. Phylogram showing the phylogenetic relationships among the 670 accession number sequences from GenBank was constructed by a Bayesian inference analysis using mitochondrial cytochrome c oxidase I (COI) sequences.

Supplementary Fig. 2. Phylogram showing the phylogenetic relationships among the 782 accession number sequences from GenBank was constructed by a Bayesian inference analysis using mitochondrial cytochrome b (Cytb) sequences.

Supplementary Fig. 3. Phylogram showing the phylogenetic relationships among the 1,518 accession number sequences from GenBank was constructed by a Bayesian inference analysis using mitochondrial D-loop sequences.

Supplementary Fig. 4. Phylogram showing the phylogenetic relationships among the 670 accession number sequences from GenBank was constructed by Bayesian inference analysis using mitochondrial cytochrome c oxidase I (COI) sequences. Group 1: higher-level of similarity with the same species. Group 2: higher-level of similarity with multiple species. Group 3: unique sequences with no similarity within most sequences. Class 1: sequences with the same species name exhibiting intraspecific cohesive clustering and interspecific distinct clustering with high posterior probability (0.90–1.00). Class 2: sequences with the same species name that do not exhibit intraspecific cohesive clustering. Class 3: sequences with a different species name exhibiting cohesive clustering. Only 1 accession number was obtained (asterisk symbol: *).

Supplementary Fig. 5. Phylogram showing the phylogenetic relationships among the 782 accession number sequences from GenBank was constructed by Bayesian inference analysis using mitochondrial cytochrome b (Cytb) sequences. Group 1: higher-level of similarity with the same species. Group 2: higher-level of similarity with multiple species. Group 3: unique sequences with no similarity within most sequences. Class 1: sequences with the same species name exhibiting intraspecific cohesive clustering and interspecific distinct clustering with high posterior probability (0.90–1.00). Class 2: sequences with the same species name that do not exhibit intraspecific cohesive clustering. Class 3: sequences with a different species name exhibiting cohesive clustering. Only 1 accession number was obtained (asterisk symbol: *).
