# Supplementary figures and images for: Overcoming taxonomic challenges in DNA barcoding for improvement of identification and preservation of clariid catfish species
Source: Genomics Inform. 2023 Sep 27;21(3):e39. doi: 10.5808/gi.23038 (PMC10584641; doi:10.5808/gi.23038)

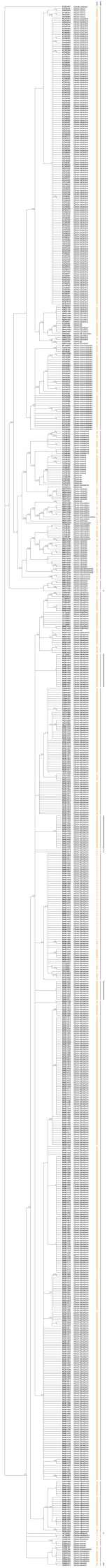

Supplement: Supplementary Fig. 2. — Phylogram showing the phylogenetic relationships among the 782 accession number sequences from GenBank was constructed by a Bayesian inference analysis using mitochondrial cytochrome b (Cytb) sequences. [file gi-23038-Supplementary-Fig-2.pdf]

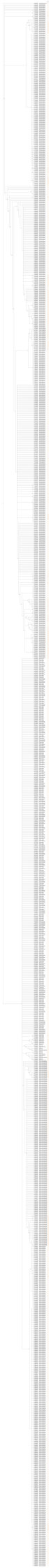

Supplement: Supplementary Fig. 3. — Phylogram showing the phylogenetic relationships among the 1,518 accession number sequences from GenBank was constructed by a Bayesian inference analysis using mitochondrial D-loop sequences. [file gi-23038-Supplementary-Fig-3.pdf]

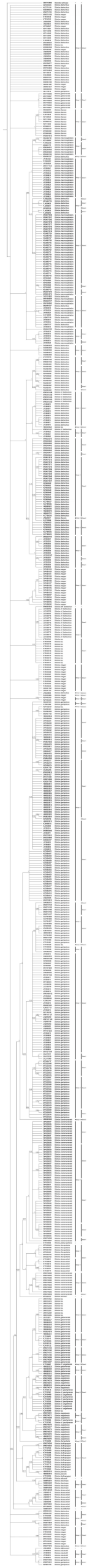

Supplement: Supplementary Fig. 4. — Phylogram showing the phylogenetic relationships among the 670 accession number sequences from GenBank was constructed by Bayesian inference analysis using mitochondrial cytochrome c oxidase I (COI) sequences. Group 1: higher-level of similarity with the same species. Group 2: higher-level of similarity with multiple species. Group 3: unique sequences with no similarity within most sequences. Class 1: sequences with the same species name exhibiting intraspecific cohesive clustering and interspecific distinct clustering with high posterior probability (0.90–1.00). Class 2: sequences with the same species name that do not exhibit intraspecific cohesive clustering. Class 3: sequences with a different species name exhibiting cohesive clustering. Only 1 accession number was obtained (asterisk symbol: *). [file gi-23038-Supplementary-Fig-4.pdf]

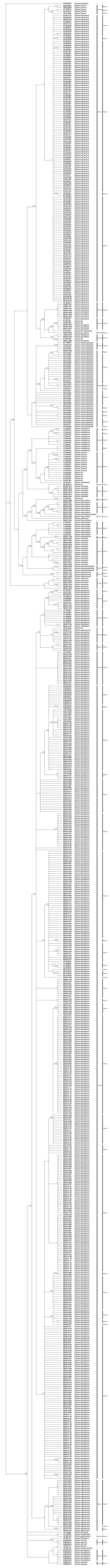

Supplement: Supplementary Fig. 5. — Phylogram showing the phylogenetic relationships among the 782 accession number sequences from GenBank was constructed by Bayesian inference analysis using mitochondrial cytochrome b (Cytb) sequences. Group 1: higher-level of similarity with the same species. Group 2: higher-level of similarity with multiple species. Group 3: unique sequences with no similarity within most sequences. Class 1: sequences with the same species name exhibiting intraspecific cohesive clustering and interspecific distinct clustering with high posterior probability (0.90–1.00). Class 2: sequences with the same species name that do not exhibit intraspecific cohesive clustering. Class 3: sequences with a different species name exhibiting cohesive clustering. Only 1 accession number was obtained (asterisk symbol: *). [file gi-23038-Supplementary-Fig-5.pdf]
