# Supplementary material for: Overcoming taxonomic challenges in DNA barcoding for improvement of identification and preservation of clariid catfish species
Source: Genomics Inform. 2023 Sep 27;21(3):e39. doi: 10.5808/gi.23038 (PMC10584641; doi:10.5808/gi.23038)

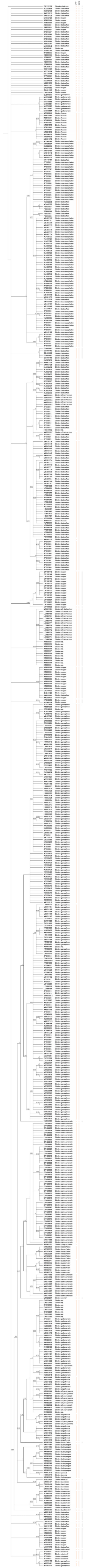

Supplement: Supplementary Fig. 1. — Phylogram showing the phylogenetic relationships among the 670 accession number sequences from GenBank was constructed by a Bayesian inference analysis using mitochondrial cytochrome c oxidase I (COI) sequences. [file gi-23038-Supplementary-Fig-1.pdf]
